# Anisotropic Grain Growth in (111) Nanotwinned Cu Films by DC Electrodeposition

**DOI:** 10.3390/ma13010134

**Published:** 2019-12-28

**Authors:** Tien-Lin Lu, Yu-An Shen, John A. Wu, Chih Chen

**Affiliations:** Department of Materials Science & Engineering, National Chiao Tung University, Hsin-chu 30010, Taiwan; tienlin308@gmail.com (T.-L.L.); r123845986@hotmail.com (Y.-A.S.); defrosticicle@gmail.com (J.A.W.)

**Keywords:** nanotwinned Cu, grain growth, preferred orientation, electrodeposition, thermal annealing

## Abstract

We have reported a method of fabricating (111)-orientated nanotwinned copper (nt-Cu) by direct current electroplating. X-ray analysis was performed for the samples annealed at 200 to 350 °C for an hour. X-ray diffraction indicates that the (200) signal intensity increases while (111) decreases. Abnormal grain growth normally results from transformation of surface energy or strain energy density. The average grain size increased from 3.8 µm for the as-deposited Cu films to 65–70 µm after the annealing at 250 °C for 1 h. For comparison, no significant grain growth behavior was observed by random Cu film after annealing for an hour. This research shows the potential for its broad electric application in interconnects and three-dimensional integrated circuit (3D IC) packaging.

## 1. Introduction

Copper has been widely used as a replacement for aluminum in the electronics industry due to its low resistivity and ideal mechanical properties [1,2]. Copper interconnects are fabricated using the damascene process where the Cu electrodeposition method is used due to its unique properties to fill in narrow trenches. However, advances in technology create a high demand in copper with high mechanical strength. Generally, methods for strengthening metals include solid solution strengthening, grain boundary strengthening, precipitation hardening, and work hardening. Recent research has also proven that another factor in increasing the mechanical strength of materials is by applying twin boundary strengthening. Nanotwinned copper (nt-Cu) structure can greatly enhance the mechanical properties and retain similar electrical properties and resistivity as that of regular Cu [3,4]. Therefore, the study of nt-Cu has become very important. In addition, Cu lines have been widely used in various electronic components, where one of the most common reliability issues is the electromigration in Cu lines. Using nt-Cu as the material of the line, it was observed that the twin structure can increase resistance against electromigration [5].

However, many reports on nt-Cu thin films claim fabrication by sputtering deposition, which is not conducive to the thickness required for industrial applications. Therefore, due to low cost and thickness control, we use an electroplating process to make nt-Cu. Hsiao et al. adapted a direct current method to fabricate high-density nt-Cu possessing a (111) oriented columnar grain structure [6]. The nanotwinned Cu microstructure with (111)-preferred orientation exhibits a single direction columnar grain, and the twin spacing ranged from several nanometers to several hundred nanometers.

In the last few decades, there has been a dramatic proliferation of research related to the mechanism of crystal growth, since it affects the properties of crystal structure in the thermal annealing process [7,8,9,10,11,12,13,14,15,16,17]. A lot of research has been done in this field to observe large grain growth [18,19,20,21,22,23,24,25,26,27,28]. The extra-large grain growth in polycrystalline Cu is called abnormal grain growth. Lu et al. reported that extremely large anisotropic grain growth occurred in (111)-oriented nanotwinned Cu films fabricated by pulsed electrodeposition [29]. The grains grew to 400 µm in a 7-µm-thick Cu film after 400 °C for 1 h. The microstructure will change dramatically, transforming from a preferred orientation of (111) to (200).

However, the annealing temperature is still too high for some applications, such as the application of manufacturing single crystal Cu on a printed circuit board (PCB) substrate [30]. In general, single crystal Cu has better thermal and electrical properties than polycrystalline Cu. (200) oriented single crystal Cu has lower resistivity and slower surface diffusion speed than other crystal faces [31,32], so it is suitable for the application of packaged bump metal pad and integrated circuit. In other words, if the temperature of the heat treatment on the process can be effectively reduced, it is extremely useful for providing circuit connections of parts on the PCB substrate. Therefore, we report a new process to reduce the heat treatment temperature for anisotropic grain growth to prepare a single crystal Cu film. Although substantial studies have been performed on the critical factors that affect abnormal grain growth of regular Cu, those of nt-Cu are still critically lacking. Our main goal is to understand the crystal growth under different annealing temperatures to better determine their excellent mechanical and electrical properties.

When the copper wire width is less than 50 nm, the resistance increases due to grain boundary scattering. If the large grain growth of the nanotwinned crystal can be utilized, we can lower the overall resistance by reducing grain boundaries. In this study, we prepare (111)-oriented nanotwinned Cu films by direct current electrodeposition. Through two-stage electroplating, we attempt to fabricate an unstable nanotwinned Cu film structure to achieve a trend of lowering thermal stability. Possible mechanisms are proposed in this paper.

## 2. Experimental Section

In order to fabricate (111)-oriented nanotwinned copper film, a 100 nm thick layer titanium barrier layer and 200 nm thick copper seed layer was sequentially sputtered on top of a silicon wafer. The Si wafer was cut into pieces of 4 × 1 cm^2^ or 3 × 1 cm^2^, and these pieces were immersed in the electrolyte during direct current electroplating. The electroplating solution used in the experiment was prepared from Cu sulfate pentahydrate crystals (CuSO_4_∙5H_2_O) with Cu cations (0.8 M), 80 ppm of chloride ions, 40 ppm hydrochloric acid, and an organic additive to assist twin formation.

Before electroplating, the pieces were immersed in citric acid to remove surface oxides, and then acetone and isopropyl were used to clean surface contaminants and organic molecules. The power supply (Keithley 2400 Source Meter, USA) was controlled by a computer to apply the current required for plating. A high-purity Cu target was placed on the anode side of the plating bath, and the pieces were placed on the cathode side of the plating bath. Deposition method was direct current electroplating, while controlling the current density to 80 mA/cm^2^ (8ASD), the stirring speed at 1200 rpm, and operating time of 240 s. To prepare randomly oriented Cu and comparison, we electroplated under the same conditions, without the additive in the electroplating solution.

The nanotwinned Cu film with a thickness of 4 microns was deposited in the first stage. And then electropolishing was performed to lower copper surface roughness for further deposition. The polished pieces then were subjected to a second stage deposition to obtain a (111)-oriented nanotwinned Cu film with total thickness of approximately 7 microns. After the plating was completed, it was cleaned with citric acid to remove surface metal oxides. Then, cleaned with deionized water and purged with nitrogen gas. Thermal annealing was carried out at 200, 250, 300, and 350 °C for 1 h in a quartz-tube furnace with a vacuum of 10^−3^ torr, electrical voltage of 120 V/60 hertz, power consumption of 800 watts, and heating rate of 0.6–1 °C/section.

X-ray diffractometer (XRD, BRUKER D2 PHASER, Karlsruhe, Germany) was used to analyze the grain orientation and peak intensity before and after the thermal annealing. The microstructure of the electrodeposited Cu film was analyzed by focus ion beam (FIB, FEI Nova 200, Brno, Czech Republic). Finally, the grain size and grain orientation after grain growth were observed by electron backscatter diffraction (EBSD, JEOL 7800F field-emission scanning electron microscope Abingdon, UK with an Oxford system).

## 3. Results and Discussion

**Figure 1:**
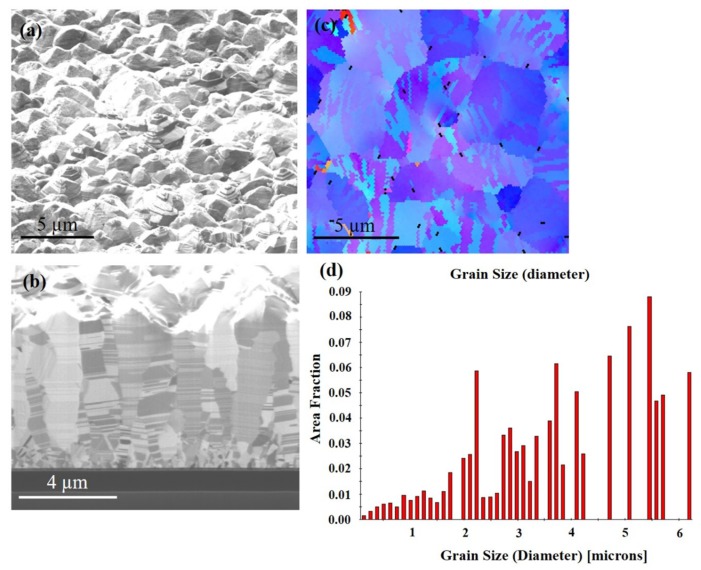
FIB micrographs of as-deposited Cu film. (**a**) Top surface. (**b**) Cross section. (**c**) Plan-view of the inverse pole figure map from electron backscatter diffraction (EBSD) of the sample at initial state. (**d**) Grain size distribution of the grains in pole Figure 1c.

Direct current electrodeposition was used to fabricate highly (111)-oriented columnar crystal grains with densely-packed nanotwinned film. Figure 1a shows the plan-view focused ion beam (FIB) image, showing the as-fabricated (111)-oriented Cu film. There are many cone structures on the surface. Figure 1b shows the cross-sectional FIB image of the Cu films. Above the seed layer is a fine-grain transition layer with a thickness of about 0.4 µm, followed by columnar grains, which are high-density nanotwins. A nanotwinned crystal is composed of a plurality of homogeneous and heterogeneous twin boundaries. Figure 1c shows the orientation image map (OIM) from electron backscattered diffraction (EBSD) for the as-fabricated Cu film and the grain size distribution for the grains is in Figure 1d. From the image, it can be seen that there is a distribution of blue and purple color. This color indicates that the crystal is (111) oriented. The average grain size is about 3.8 ± 1 µm.

**Figure 2:**
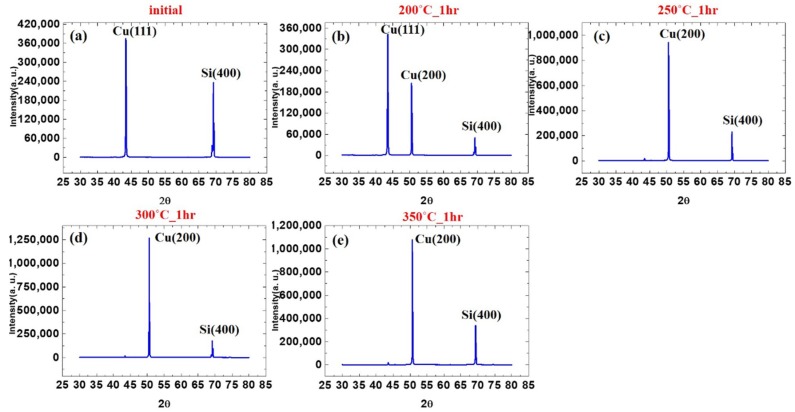
X-ray diffraction intensities of the samples at (**a**) Initial state and annealed at (**b**) 200 °C. (**c**) 250 °C. (**d**) 300 °C. (**e**) 350 °C for an hour.

**Figure 3:**
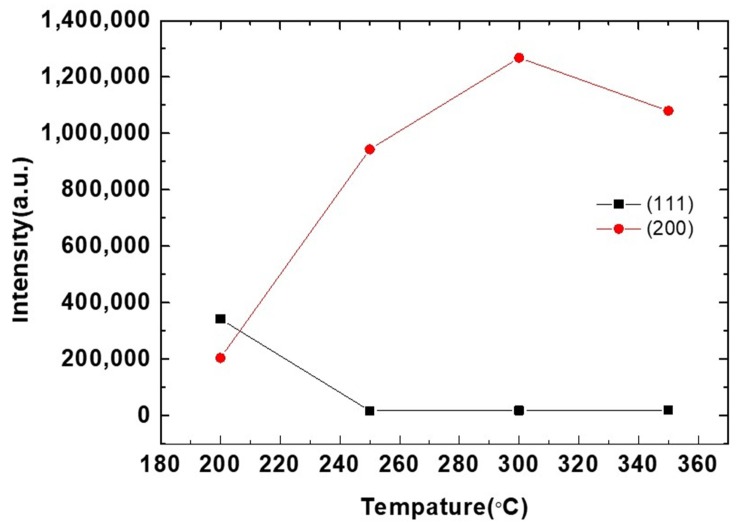
Comparison of (111) and (200) intensities with annealing temperature.

In order to observe the transformation of each stage after annealing, experiments were carried out at different temperatures. Figure 2a–e shows the evolution of X-ray diffraction spectra for the Cu films at the initial state, annealed at 200, 250, 300, 350 °C for an hour. A very strong (111) preferred orientation was observed in the initial state by XRD analysis. Figure 2a shows that the intensity ratio of I_(111)_/I_(200)_ is as high as 261. The electroplated nanotwinned Cu has extremely high (111) texture. However, it is observed in Figure 2b that (200) preferred orientation appears after one hour of annealing at 200 °C. A very strong (200) preferred orientation was observed after annealing at 250 °C from Figure 2c, which was significantly stronger than the (111) preferred orientation with a I_(200)_/I_(111)_ ratio of 56. As the annealing temperature continues to increase to 300–350 °C, the peak intensity of (200) further increased, as illustrated in Figure 2d,e. As the annealing temperature increases, the preferred orientation of (111) decreases and the orientation of (200) increases. The results are organized graphically in Figure 3. From the curve in the figure, it can be observed that when the sample is annealed at 250 and 350 °C, there is an obvious anisotropic growth of (100) grains.

**Figure 4:**
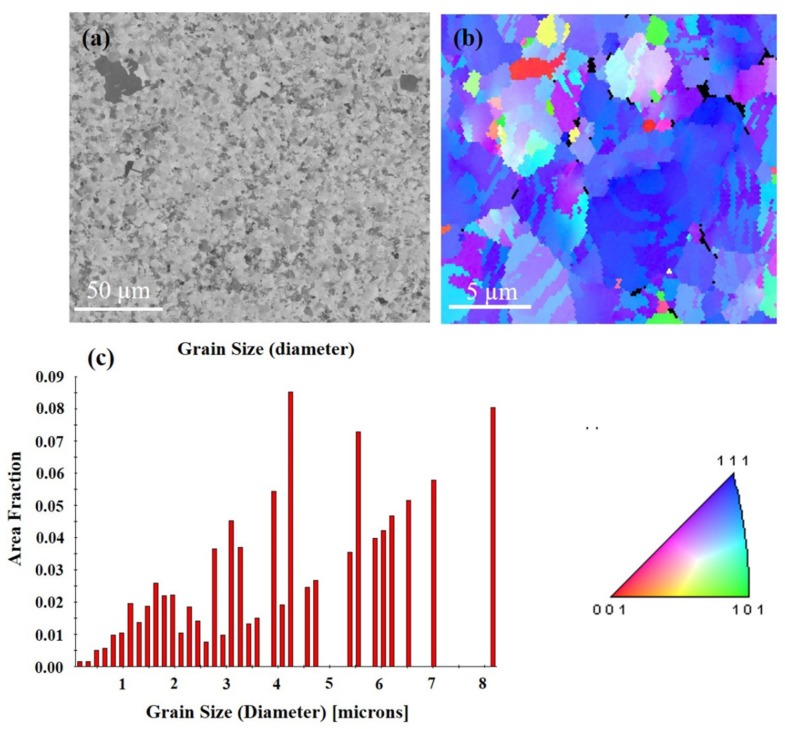
Grain morphologies for the Cu film at 200 °C for an hour. (**a**) FIB top-view image. (**b**) EBSD orientation image. (**c**) Grain size distribution of the grains in pole Figure 4b.

To observe the effect of annealing on the surface structure of the Cu film, we compare the EBSD results of the different films. Figure 4a,b presents the FIB top-view image and the plan-view of the orientation image map from EBSD of the Cu film annealed at 200 °C for an hour. Compared with Figure 1 of the initial state, it was observed that the surface grains began nucleation and the grain size increased. Figure 4c depicts the grain size distribution of the grains. There is no significant difference in average grain size between 200 °C and initial conditions. Only a few grains begin to nucleate into large grains.

**Figure 5:**
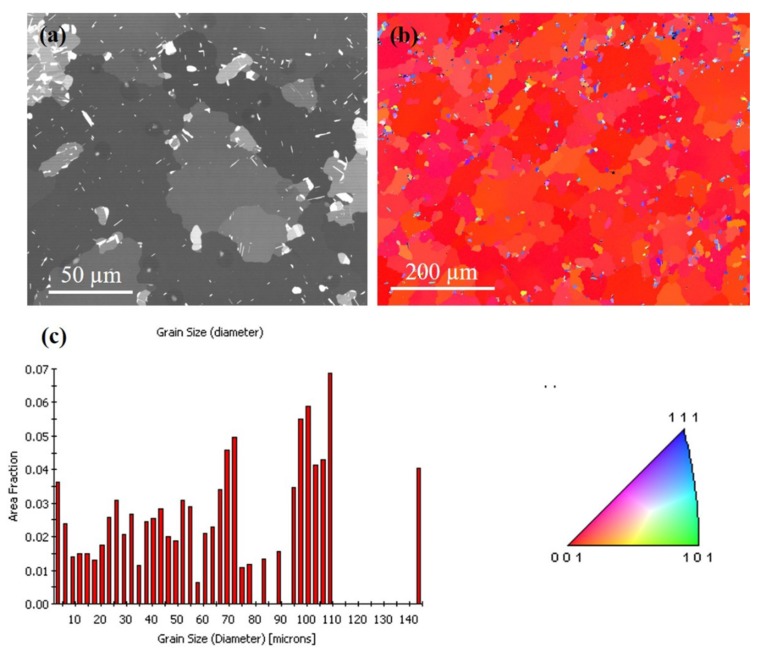
Grain morphologies for the Cu film at 250 °C for an hour. (**a**) FIB top-view image. (**b**) EBSD orientation image. (**c**) Grain size distribution of the grains in pole Figure 5b.

**Figure 6:**
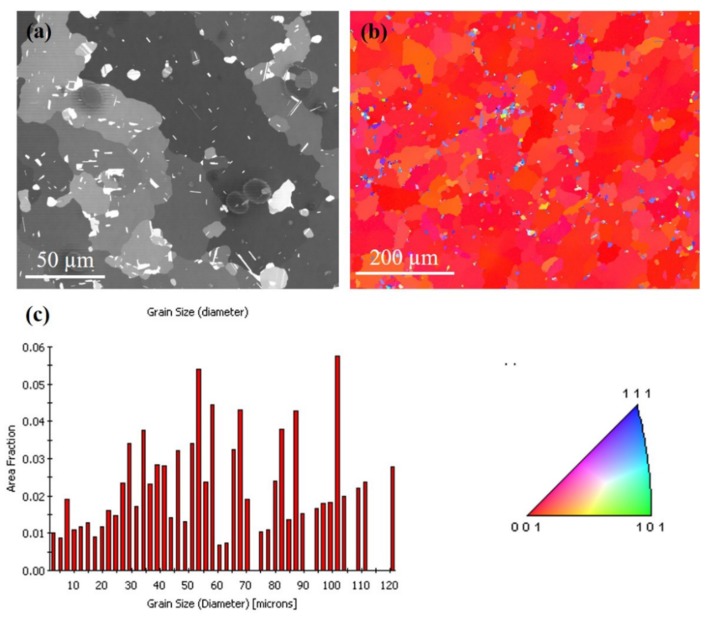
Grain morphologies for the Cu film at 300 °C for an hour. (**a**) FIB top-view image. (**b**) EBSD orientation image. (**c**) Grain size distribution of the grains in pole Figure 6b.

**Figure 7:**
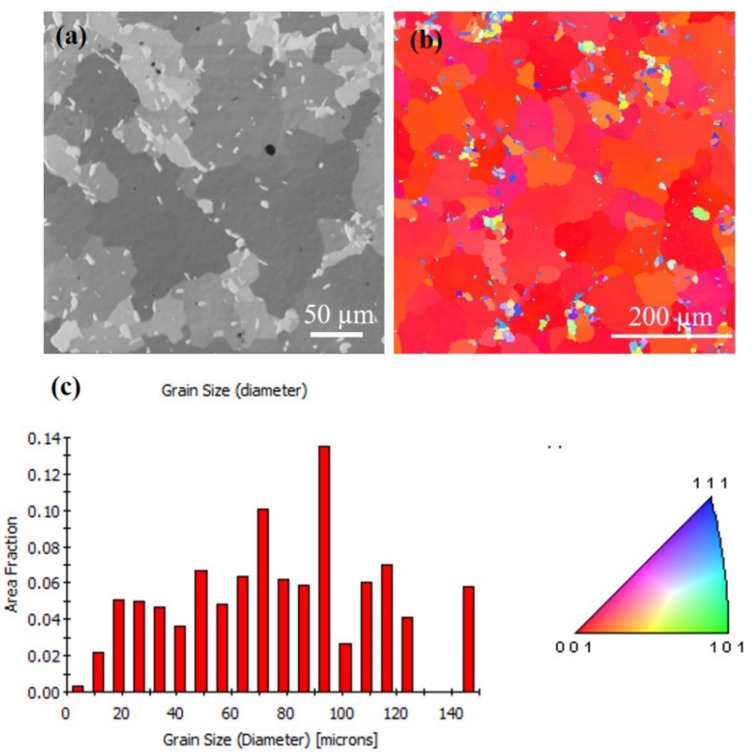
Grain morphologies for the Cu film at 350 °C for an hour. (**a**) FIB top-view image. (**b**) EBSD orientation image. (**c**) Grain size distribution of the grains in pole Figure 7b.

**Figure 8:**
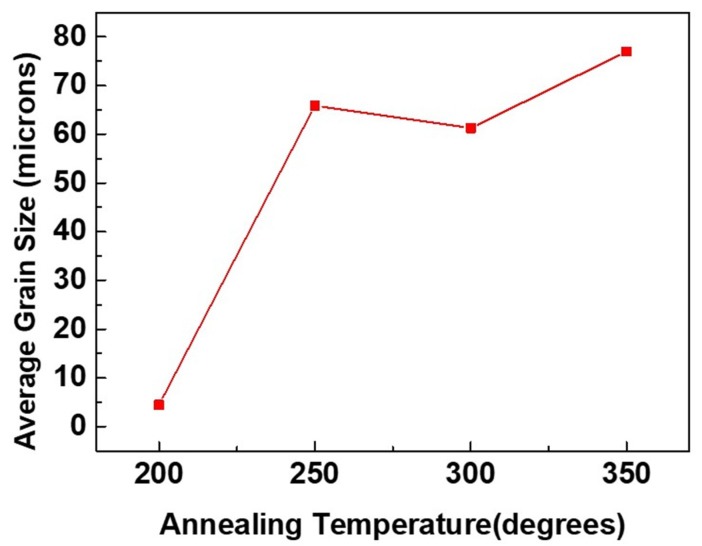
Average grain size variation under various annealing conditions.

The FIB grain image for the sample annealed at 250 °C for an hour is depicted in Figure 5a. The largest grain is approximately 20 times larger than the surrounding grains. Figure 5b shows the orientation image map from EBSD for the grain on the top surface, indicating that most of the crystal orientation is (200) oriented. The grain size distribution of the grains is in Figure 5c. There was large grain growth on the surface, and the average grain size increased from 3–4 µm (as-deposited) to 65–70 µm (250 °C). When the <111> preferred orientation of nt-Cu film is annealed, the surface microstructure will change from the preferred orientation of (111) to (100) large grains. It is speculated that the behavior of anisotropic grain growth may be caused by structural instability caused by two-stage electroplating. Furthermore, an increase in surface grain size was observed with increased annealing temperature. Figure 6a,b present top-view FIB and plan-view EBSD images, respectively, for the abnormal grain growth of (200) grain annealed at 300 °C for an hour. This average grain size is approximately 61 µm and distribution of the grains are in Figure 6c. Figure 7a depicts the FIB image for the Cu film annealed at 350 °C. The EBSD grain morphology on the top surface and the width of the average grain was approximately 77 µm as shown in Figure 7b,c. The average grain size changes after annealing at different temperatures are shown in Figure 8. Abnormal grain growth is observed between 200 and 250 °C, and the average grain size tends to increase when the temperature is higher.

According to the XRD (111) and (200) intensity ratios shown in Figure 3, there is a huge preferred orientation change after annealing at over 250 °C for 1 h. The nt-Cu indicating a strong (111) preferred orientation disappeared after annealing and converted into (200) large single crystal Cu. This is reflected in the EBSD results. Grains convert to (200) large grains after annealing over 250 °C. Abnormal grain growth of the (200) large grains can be easily observed in the sample annealed from 250 to 350 °C as seen in Figure 4, Figure 5, Figure 6 and Figure 7. There are many factors affecting the size and distribution of grain, such as surface energy inside the grain, residual stress of the film, grain boundary diffusion, and recrystallization, etc. [33,34]. Among them, the influence of surface energy is the most critical factor. It is presented in research that the surface energy is related to the grain size of the film itself [35]. Therefore, the reason for the uneven distribution of grain size is attributed to the number of nucleation. The more the nucleation that occurs, the smaller the grain size. We measured Cu films at initial pre-annealing and different annealing temperatures of 200, 250, 300, and 350 °C. The calculated resistivity is 2.07, 2.02, 1.99, 1.96, 1.89 (µΩ-cm) for as-deposited and the listed annealing temperatures. It can be observed that the annealing temperature has little effect on the resistivity, but it does tend to decrease slightly. Since the electrical conductivity of a metal is determined by its resistivity, the lower the resistivity, the lower the voltage drop under the same currents, and the relative power consumption is smaller.

Annealing has a great influence on the microstructure of Cu films. Past research literatures [9] indicate that the preferred orientation of Cu grains is determined according to the competition of energy after annealing treatment. The lowest interface energy or surface energy of face-centered cubic (FCC) structures is (111), and the lowest strain energy is (100). Since there is a mismatch between the coefficient of thermal expansion of the substrate and the Cu film, the existence of thermal stress during the annealing process leads to an increase in strain energy, so the crystal grains grow rapidly after nucleation of the Cu film. Therefore, from the experiments, we observe that the microstructure of the Cu film depends on the annealing temperature. At lower annealing temperatures, the preferred orientation of the Cu film tends to remain (111)-preferred, and conversely at the higher annealing temperatures, the orientation of the Cu film tend to be (100)-preferred. The annealing temperature plays a crucial role in the abnormal grain growth of nt-Cu. We have observed that (111) grains convert to (100) grains after annealing at 250 °C for one hour, with abnormal grain growth occurring across nearly the entire surface, as shown in Figure 5, Figure 6 and Figure 7. Therefore, we speculate that 250 °C is the critical temperature for abnormal grain growth to take place. Annealing under the critical temperature drives the consumption of the fine grain region at the bottom of the films, while rising above the critical temperature causes nucleation and grain growth.

**Figure 9:**
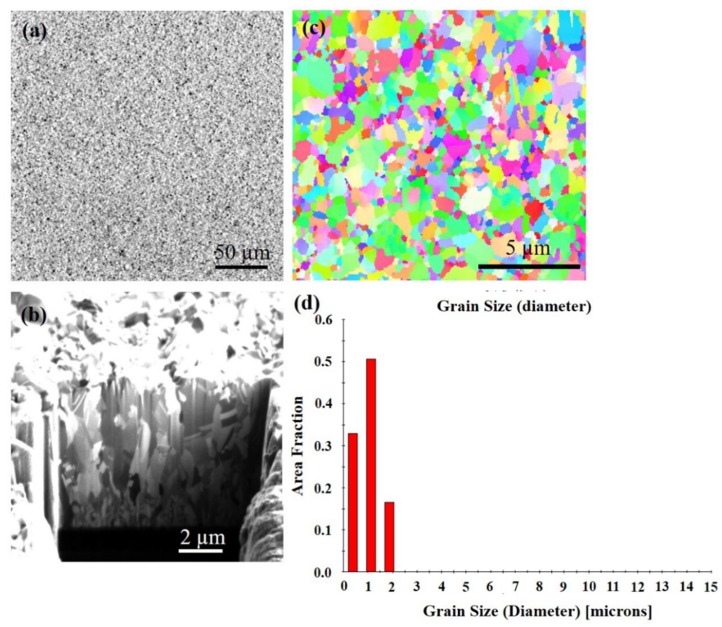
FIB micrographs of randomly oriented Cu. (**a**) Top surface. (**b**) Cross section. (**c**) Plan-view of the inverse pole figure map from EBSD of the sample at initial state. (**d**) Grain size distribution of the grains in pole Figure 9c.

**Figure 10:**
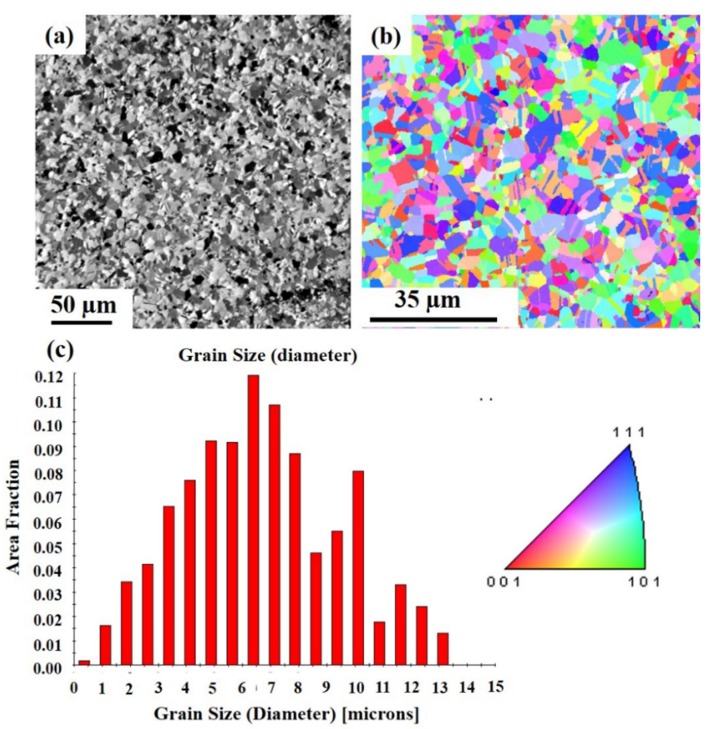
Normal grain growth of randomly oriented Cu films at 350 °C for an hour. (**a**) FIB top-view image. (**b**) EBSD orientation image. (**c**) Grain size distribution of the grains in pole Figure 9b.

To compare our results, we prepared randomly oriented Cu to observe grain growth behavior. Figure 9a presents a plan-view focused ion beam image of randomly oriented Cu. Figure 9b shows the cross-sectional FIB image of the randomly oriented Cu films, showing random crystal orientation, and no twin crystal formation. Figure 9c shows the orientation image map from electron backscattered diffraction (EBSD) for the pre-annealed state and the grain size distribution for the grains is in Figure 9d. The plan view of the surface appears as a messy crystal orientation with an initial average grain size of about 1.01 μm. However, no extremely abnormal grain growth was observed after the thermal annealing. Figure 10a shows the top-view FIB images of the randomly oriented Cu film annealed at 350 °C for an hour, normal grain growth occurred. The EBSD grain morphology on the top surface and the width of the average grain size was only 6.66 µm as shown in Figure 10b,c, which is approximately the thickness of the Cu film.

We compared the evolution of nt-Cu with (111)-oriented and randomly oriented Cu after annealing. During the heat treatment process of (111)-oriented nt-Cu at 250–350 °C, the microstructure changes greatly, and the behavior of abnormal grain growth transforms into (200) large single crystal Cu over a certain annealing temperature. It is speculated that the possible reason is that the polishing after the first stage flattens the surface and continued plating of the second stage causes instability of the structure. The polishing after the first stage flattens the surface and continued plating causes instability of the structure. During the two-step electrodeposition, there will be a short period of non-current time between the two electroplating processes, similar to the off-time in pulse electrodeposition. This off-time like period allows strain relaxation in the thin film causing strain energy decrease in the nt-Cu structure [36,37]. This is a reason why abnormal grain growth occurs after the annealing process. Therefore, we have discovered that such an unstable structure can greatly change the microstructure of the nt-Cu film when it is annealed at a low temperature of 250 °C, which will become (100) oriented large grains.

## 4. Conclusions

After two-stage electroplating, our results show that Cu (111) converts into Cu (200) after annealing at over 250 °C. We were able to fabricate highly (111)-oriented Cu grains with densely packed nanotwins by direct current electroplating. We observed only (111) and (200) signals, with the intensity of the former decreases while the latter increases with the increase of annealing temperature. Abnormal grain growth results in texture transformation under surface energy or strain energy density change. On the other hand, the two-stage electroplated nanotwinned crystal disappeared after low temperature annealing, and the thermal stability was lowered. We can effectively reduce the temperature required for abnormal grain growth. 

For the low temperature process in the industry, the temperature of the heat treatment can be effectively reduced to prepare (200) oriented single crystal Cu, which is of great value for the application of printed circuit board coating. From the structural point of view, Cu (200) may be better than Cu (111) in resisting electromigration.
